# Preparation, Adsorption Performance and Mechanism of Low-Cost Desert Sand-Based Pb (II) Ion-Imprinted Composites

**DOI:** 10.3390/polym18010042

**Published:** 2025-12-23

**Authors:** Yixin Sui, Jiaxiang Qi, Shuaibing Gao, Linlin Chai, Yahong Xie, Changyan Guo, Shawket Abliz

**Affiliations:** Key Laboratory of Oil and Gas Fine Chemicals of Ministry of Education, School of Chemical Engineering, Xinjiang University, Urumqi 830017, China

**Keywords:** desert sand, ion-imprinted composites, Pb (II), adsorption performance, selectivity, reusability

## Abstract

Pb (II) contamination in wastewater represents a grave threat to the environment and ecosystems. Consequently, there is an urgent need to prepare low-cost and highly efficient Pb (II) adsorbents. To address this need, abundant and low-cost natural silica-based desert sand (DS) was innovatively utilized as a carrier to develop efficient and selective Pb (II) adsorbents. Modified desert sand (MDS) was first prepared via 1 M HCl pretreatment for 2 h and subsequent KH550 silane modification. Pb (II)-imprinted composites (Pb (II)-IIP@MDS) were then fabricated via ion-imprinted polymerization, using Pb (II) as the template ion and N-hydroxymethacrylamide (NHMA)/hydroxyethyl methacrylate (HEMA) as dual functional monomers with a molar ratio of 1:1. The synthesized Pb (II)-IIP@MDS was comprehensively characterized by X-ray photoelectron spectrometer (XPS), scanning electron microscopy (SEM), and Fourier transform infrared spectroscopy (FT-IR). The adsorption capacity, selectivity, and reusability of this material for lead ions were evaluated through three experiments conducted within the optimized pH range of 6–7, with error bars indicated. In adsorption isotherm experiments, the initial Pb (II) concentration ranged from 50 to 500 mg·L^−1^, conforming to the Langmuir model (R^2^ = 0.992), with a theoretical maximum adsorption capacity reaching 107.44 mg·g^−1^; this indicates that the adsorbate forms a monolayer adsorption on the homogeneous imprinted sites. Kinetics data indicate that the process best fits a quasi-first-order kinetic model (R^2^ ≥ 0.988), while the favorable quasi-second-order kinetic fit (R^2^ ≥ 0.982) reflects the synergistic effect of physical diffusion and ion-imprinting chemistry, reaching equilibrium within 120 min. Thermodynamic parameters (ΔH^0^ = 12.51 kJ·mol^−1^, ΔS^0^ = 101.19 J·mol^−1^·K^−1^, ΔG^0^ < 0) confirmed endothermic, entropy-increasing, spontaneous adsorption. In multicomponent systems, Pb (II)-IIP@MDS showed distinct Pb (II) selectivity. It retained 80.3% adsorption efficiency after eight cycles. This work provides a promising strategy for fabricating low-cost, high-performance Pb (II) adsorbents, and Pb (II)-IIP@MDS stands as a practical candidate for the remediation of Pb (II)-contaminated wastewater.

## 1. Introduction

Lead (Pb (II)) is a representative non-biodegradable, bioaccumulative toxic heavy metal in the environment [1], with the World Health Organization (WHO) setting its maximum allowable concentration in drinking water at 10 μg·L^−1^ [2]. Owing to its non-biodegradability and bioaccumulation tendency, it poses long-term risks to ecosystems and human health [3]. Sources of contamination include industrial emissions (mineral smelting, lead-acid battery production, metal electroplating), traditional printing ink applications, and dismantling of waste electronic equipment [4,5,6]. Uncontrolled discharge of Pb (II)-containing wastewater disrupts ecological balance through soil permeation and water migration. Its bioaccumulation in humans via the food chain can cause severe complications, including childhood cognitive developmental disorders, neurological diseases, kidney damage, and cardiovascular abnormalities [7,8,9,10,11]. Consequently, developing highly efficient remediation technologies for Pb (II)-contaminated water bodies has become an urgent necessity in water environmental restoration.

Presently, the main treatment technologies for aqueous Pb (II) include chemical precipitation [12,13], electrolysis [14,15,16], adsorption [17,18], and membrane separation [19,20,21]. Among these, chemical precipitation is easy to operate but tends to generate large amounts of lead-containing sludge, causing secondary pollution [22]. Electrolysis and membrane separation, however, suffer from high cost and energy consumption, making them challenging for large-scale treatment of low-concentration Pb (II) wastewater [23]. In contrast, adsorption has emerged as the mainstream technology in current research and application due to its advantages of simple equipment, high efficiency, and tunable selectivity [24]. However, conventional adsorbents (e.g., activated carbon, zeolites, and biomass charcoal) primarily rely on non-specific physical adsorption or elementary chemical interactions [25]. Certain materials exhibit insufficient selectivity toward Pb (II) in complex water systems, are susceptible to interference from coexisting heavy metal ions, and feature low adsorption capacity and poor regenerative capability, thereby limiting their practical application in wastewater treatment [26,27].

To address the selectivity bottleneck of traditional adsorbents, ion-imprinting technology (IIT)—a sophisticated adsorption strategy that constructs recognition sites on adsorbents to match target ions—has garnered significant attention [28]. This technology comprises three core steps, thereby enabling selective binding of target ions relative to interfering ions [29]: pre-complexation of template ions with functional monomers, cross-linked polymerization to form a polymer matrix, and template elution to generate specific imprinted cavities with “size-charge-configuration matching”.

In recent years, scholars worldwide have conducted extensive research on Pb (II) ion-imprinted materials (IIPs), using synthetic carriers (e.g., silica gel, mesoporous MCM-41, metal–organic frameworks (MOFs)) and established functional monomers (e.g., methacrylic acid (MAA), acrylamide (AAm), 4-vinylpyridine (4-VP)) [28,30,31]. Some of these materials exhibit Pb (II) adsorption capacities of 40–120 mg·g^−1^, with selectivity coefficients significantly higher than those of non-imprinted counterparts [32,33,34]. Zhang X. et al. [35] prepared multi-walled carbon nanotubes (MWCNTs)-based Pb (II) ion-imprinted polymers (IIPs), which exhibited a maximum adsorption capacity of 18.09 mg·g^−1^ for Pb (II) at the optimal adsorption temperature of 45 °C. Even the magnetic ion-imprinted membrane (MIIM) with magnetic separation and antibacterial properties achieves only 45.4 mg·g^−1^ selective adsorption capacity for Pb (II) in coexisting ion systems [36], indicating room for improvement in existing Pb (II) ion-imprinted materials.

It is important to acknowledge that existing Pb (II) ion-imprinted materials continue to encounter the following limitations, which are indicative of the fundamental scientific gaps that this study aims to address: The initial observation is that the range of carriers is limited. The majority of studies use synthetic nanocarriers (e.g., MCM-41, MOFs), whose preparation involves high costs and complex processes, thus posing significant challenges for large-scale production. These issues complicate the achievement of an equilibrium between performance and cost-effectiveness in the context of practical wastewater treatment [28,30]. Secondly, the material exhibits inadequate stability. Research has demonstrated that specific organic polymer-imprinted materials are susceptible to dissolution or structural degradation under acidic desorption conditions, leading to suboptimal reusability [31]. Thirdly, the efficiency of monomer coordination is inadequate. Conventional monomers generally depend exclusively on a single functional group for binding Pb (II), leading to restricted template ion affinity and inadequate selectivity in multi-ion systems [34]. Consequently, the development of Pb (II) ion-imprinted materials using cost-effective natural carriers, highly stable silicon-based frameworks, and multifunctional monomer systems has emerged as a pivotal area of focus and a significant challenge in contemporary research within this field.

It is noteworthy that few studies have reported on sand particles as carriers for ion-imprinted materials. Desert sand (DS) constitutes an abundant, inexpensive, and readily available natural silica-based carrier, primarily composed of silica [37]. The material displays both excellent chemical stability and mechanical strength [38], with a surface rich in hydroxyl (-Si-OH) active sites that promote the graft polymerization of functional monomers [39]. The granular structure of sand particles reduces preparation costs, while the silicon-based framework enhances stability. Functional groups introduced through surface modification can serve as recognition sites for constructing imprint cavities, thereby offering a highly promising solution to the issues of high cost and insufficient stability in ion-imprinted materials.

To tackle the aforementioned issues, DS was utilized in this work, with its surface structure optimized through a two-step modification process: hydrochloric acid pretreatment to eliminate impurities and activate surface hydroxyl groups, followed by KH550 silane modification to introduce amino groups that enable efficient grafting of functional monomers. Subsequently, Pb (II)-imprinted polymers (denoted as Pb (II)-IIP@MDS, with IIP referring to ion-imprinted polymer and MDS to modified desert sand) were synthesized using Pb (II) as the template ion, ethylene glycol dimethacrylate (EGDMA) as the cross-linking agent, and potassium persulfate (KPS) as the initiator. This study encompasses two key innovations: first, the employment of DS as a low-cost natural carrier to resolve the high cost of synthetic carriers, and second, the adoption of dual functional monomers N-hydroxymethacrylamide (NHMA) and hydroxyethyl methacrylate (HEMA), where the synergistic coordination of -CONH- and -OH boosts the template-binding capacity and selectivity. Unlike mature monomers such as MAA, AAm, or 4-VP, which rely on forming monodentate coordination bonds with Pb (II), the dimer combination of NHMA and HEMA enables bidentate cooperative chelation through the -CONH- and -OH groups, thereby achieving higher affinity and interference resistance selectivity [40].

This study hypothesizes that MDS can serve as a stable, low-cost carrier and that the dual-monomer system enhances affinity for Pb (II). Its core objective is to develop a high-performance, cost-effective Pb (II)-imprinted adsorbent for wastewater treatment.

## 2. Experimental Section

### 2.1. Chemical Reagents

All chemicals utilized in this study were of analytical grade. Potassium persulfate (KPS) was acquired from Tianjin Zhiyuan Chemical Reagent Co., Ltd. (Tianjin, China); N-hydroxymethacrylamide (NHMA, purity 98%) and ethylene glycol dimethacrylate (EGDMA, purity 98%) were purchased from Shanghai Macklin Biochemical Co., Ltd. (Shanghai, China); anhydrous ethanol was supplied by Tianjin Xinbote Chemical Co., Ltd. (Tianjin, China); 3-aminopropyltriethoxysilane (KH550) and hydroxyethyl methacrylate (HEMA, purity 96%, containing ppm-level MEHQ as stabilizer) were acquired from Shandong Keyuan Biochemical Co., Ltd. (Heze, China); hydrochloric acid and lead nitrate were purchased from Urumqi Dicheng Chemical Co., Ltd. (Urumqi, China); and desert sand (DS) was randomly collected from the edge area of the Taklamakan Desert, Xinjiang Uygur Autonomous Region, China. All experimental solutions were prepared using deionized water, and all material cleaning procedures during the experiment were also performed with deionized water. To achieve quantitative atomic absorption spectrophotometric analysis of Pb (II), all calibration solutions were prepared using the same high-purity Pb(NO_3_)_2_ (99%). The concentration accuracy was validated by the linear correlation coefficient (R^2^ ≥ 0.999) of the calibration curve.

### 2.2. Composite Material Preparation

#### 2.2.1. Modification of Carrier Sand Particles

Desert sand (DS) was first passed through a 200-mesh sieve (aperture 0.076 mm) to remove large solid impurities. It was then repeatedly rinsed with deionized water until the rinse solution became clear. Magnetic impurities were subsequently removed by magnetic stirring at 500 rpm for 2 h (with the rotor removed and cleaned every 15 min). After stirring, the sand was dried at 90 °C for 5 h in a drying oven, cooled to room temperature (25 ± 2 °C), and immersed in 1M hydrochloric acid solution at a solid-to-liquid ratio of 10:1 (by volume) for 2 h to remove metal oxides [40,41]. It was then rinsed again with deionized water until the washing solution pH reached 7.0 ± 0.2, followed by another 5 h of drying at 90 °C to complete pretreatment. For modified sand particle (MDS) preparation, we first dissolved 5% (*w*/*w*) KH550 in an ethanol–water mixture (9:1 *v*/*v*), adjusted pH to 4.5 with acetic acid, then added pretreated DS (liquid–solid ratio = 8:1 *v*/*v*) and impregnated for 6 h (40 °C, 300 rpm); this was followed by three washes with anhydrous ethanol to remove unreacted KH550. Finally, the mixture was dried in an 80 °C oven for 6 h to obtain MDS.

#### 2.2.2. Preparation of Template Ion/Functional Monomer Comple

At room temperature, dissolve 0.50 g of lead nitrate in 25 mL of deionized water. The molar ratio of Pb (II) to the monomer (NHMA + HEMA) was determined to be 1:4, with the NHMA:HEMA molar ratio set at 1:1. Following this optimized ratio, after complete dissolution, add 0.30 g of NHMA under continuous stirring (400 rpm). Separately, dissolve 0.35 mL HEMA in 25 mL ethanol and mix (preventing HEMA aggregation; completely miscible with water). Subsequently combine the two solutions and continue stirring for 20 min (400 rpm) to form 50 mL of stable Pb (II)/NHMA/HEMA complex. Perform UV characterization on the complex.

#### 2.2.3. Preparation of Imprinted and Unimprinted Composites

Transfer the Pb (II)/NHMA/HEMA composite to a round-bottom three-neck flask containing 5.0 g MDS and sonicate for 10 min to ensure thorough mixing. Using Pb(NO_3_)_2_ as the base, add EGDMA (cross-linker) and KPS (initiator) at a molar ratio of 1:20:0.24, mix thoroughly, degas with nitrogen for 15 min, and react at 70 °C under nitrogen atmosphere for 7 h. After reaction, wash three times with anhydrous ethanol at room temperature to remove unreacted monomers. Elute Pb (II) from the template by immersing in 0.5 M hydrochloric acid for 2 h, with elution efficiency verified by AAS until no Pb (II) signal is detected in the eluate. Wash with deionized water until neutral, vacuum filter, and dry at 50 °C to obtain Pb (II)-IIP@MDS. The non-imprinting material (Pb (II)-NIP@MDS) was prepared using the same procedure but without the addition of Pb (II).

### 2.3. Characterization

X-ray diffraction (XRD, XRD-6000, Bruker, Karlsruhe, Germany) was used for crystal structure analysis of DS and MDS (scan range: 5 < 2θ < 80°). Ultraviolet–visible (UV-Vis) spectroscopy (T6 New Century, Beijing Puxi Gen. Instr. Co., Ltd., Beijing, China) characterized relevant pre-preparation systems over 190–600 nm. Surface hydrophobicity/hydrophilicity was evaluated via an optical contact angle goniometer (OCA 25, Beijing Aodelinuo Instr. Co., Ltd., Beijing, China) using the sessile drop method (drop volume: 2 μL, ambient temperature 25 °C). Fourier transform infrared (FTIR) spectroscopy (INVENIO R, Bruker, Germany) analyzed functional groups with a 400–4000 cm^−1^ scan range (spectral resolution: 4 cm^−1^). Morphology was observed by field-emission scanning electron microscopy (SEM, Zeiss Gemini Sigma 300, Carl Zeiss AG, Oberkochen, Germany) coupled with energy-dispersive X-ray spectroscopy (EDS) for surface elemental composition and distribution. Specific surface area (Brunauer–Emmett–Teller, BET) and pore size distribution (Barrett–Joyner–Halenda, BJH) were determined using a specific surface area/pore size analyzer (V-Sorb 2800TP, Guoyi Quantum Technol. (Hefei) Co., Ltd., Hefei, China) (adsorption gas: N_2_ at 77 K, method: multipoint). Thermal stability was assessed via a thermogravimetric–differential thermal analyzer (TG-DTA, HITACHI STA7300, Hitachi Anal. Instr. (Shanghai) Co., Ltd., Shanghai, China). X-ray photoelectron spectroscopy (XPS, ESCALAB 250Xi, Thermo Fisher Scientific, Waltham, MA, USA) analyzed elemental chemical states and electron binding energy changes to investigate adsorption mechanisms. The test used Al Kα radiation (1486.6 eV), with pass energy 20 eV (narrow scan) and 100 eV (wide scan) and charge correction via C 1s (284.8 eV).

### 2.4. Adsorption Performance Tests

The adsorption of Pb (II) by Pb (II)-IIP@MDS and Pb (II)-NIP@MDS was experimentally tested, with each set of experiments repeated three times to ensure reproducibility. Their adsorption performance was evaluated under varied conditions: adsorbent dosage of 50 mg, contact time of 0–150 min, pH 1–10 (adjusted with 0.1 mol·L^−1^ HCl/NaOH), initial concentration of 50–500 mg·L^−1^, and temperature of 298–313 K (controlled by a constant-temperature water bath). We determined the residual Pb (II) concentration at 283 nm using an atomic absorption spectrometer (AAS) and calculated the adsorption amount (Q_e_) [42].

(1) $$Q_{e}=\left(C-C_{e}\right)V/m\;$$

Herein, *C* and *C_e_* denote the initial and equilibrium concentrations of Pb (II) (mg·L^−1^), respectively; *V* is the volume of the Pb (II) solution (L); and *m* is the mass of the adsorbent (g).

### 2.5. Evaluation of Selective Adsorption Performance

Selective adsorption experiments of Pb (II)-IIP@MDS and Pb (II)-NIP@MDS towards multiple metal ions were conducted. A binary “Pb-single-component” competitive system (10 mL) was prepared, containing 100 mg·L^−1^ Pb (II) and 5 mL each of 100 mg·L^−1^ other single heavy metal ions. A “Pb-all-component” competitive system (10 mL) was simultaneously prepared containing 100 mg·L^−1^ Pb (II) and equal volumes and concentrations of other heavy metal ions. An amount of 50 mg of Pb (II)-IIP@MDS and Pb (II)-NIP@MDS were added to the binary and multicomponent systems, respectively, for 120 min of adsorption. The distribution ratio, selectivity coefficient, and relative selectivity coefficient were calculated by measuring the residual Pb (II) concentration to quantitatively analyze the selectivity differences between Pb (II) and other metal ions [28]. Relevant parameters are shown in Table S5.

### 2.6. Cyclic Utilization Evaluation

0.5 M HCl was selected as the desorbent to analyze the reusability of Pb (II)-IIP@MDS over eight adsorption–desorption cycles. An amount of 50 mg of Pb (II)-IIP@MDS was added to 10 mL of 100 mg·L^−1^ Pb (II) solution for adsorption [43], which was performed at room temperature and pH 7. The adsorbent was then fully desorbed in the desorbent for 3 h (desorption completeness verified via AAS of the supernatant), dried, and reused for adsorption, repeating the cycle eight times.

For environmental safety considerations, used Pb (II)-IIP@MDS adsorbents (loaded with Pb (II)) were be collected, sealed, and transferred to a specialized hazardous waste treatment facility for centralized disposal in accordance with local environmental regulations.

## 3. Results and Discussion

### 3.1. Analysis of Material Characterization Results

#### 3.1.1. XRD Characterization

In Figure S1a, characteristic diffraction peaks of DS and HCl-treated DS (021), (130), and (112) planes align with PDF card No 83-1828 (silica standard, ICDD), confirming silica as the primary phase and HCl treatment as preserving core crystal structure. This treatment retains carrier rigidity/stability while eliminating impurities and tuning surface properties, maintaining ion-imprinted layer spatial structure and imprinted site configuration to ensure consistent target ion recognition across cycles. Enlarged key peaks in Figure S1b show differences in width and symmetry between DS and HCl-treated DS, indicating surface micro-defects induced by HCl. These defects enhance surface roughness and specific area, expose additional Si-OH active sites, and provide anchors for functional monomer grafting/polymerization.

#### 3.1.2. UV–Vis Characterization

UV absorption reflects is suggestive of binding of monomers and templates during ion-imprinting preparation. Figure S2a shows the smoothest absorption decline at the 1:4 ratio, which implies effective binding. Figure S2b shows that the 1:4 curve exhibits a uniform gradient, avoiding coordination defects and efficiency loss while balancing both. The dual monomer ratio modulates copolymer structure and electronic properties; the 1:1 ratio balances electronic effects and spatial configuration. The -CONH- group of NHMA and the -OH group of HEMA are likely to synergistically interact via hydrogen bonds/dipole interactions, altering conjugation/polarity. Figure S2c displays maximum absorbance and distinct peaks, validating that the formation of ordered copolymer precursors is crucial for imprinting cavities. Figure S2 shows that the coordination interactions after mixing monomers with Pb (II) alter monomer electron density; monomer-ion aggregates reduce absorbance, causing red shifts. Spectral synergistic changes confirm chemical coordination and the formation of a new system.

#### 3.1.3. Contact Angle Analysis

**Figure 1:**
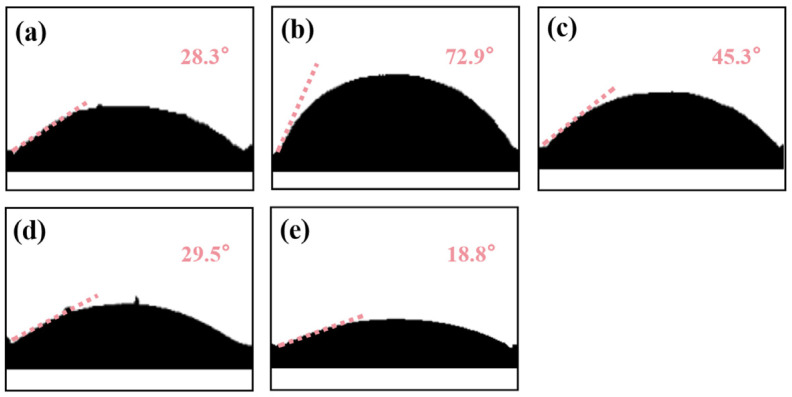
(**a**) Contact angle test results of DS; (**b**) contact angle test results of MDS; (**c**) contact angle test results of Pb (II)-NIP@MDS; (**d**) contact angle test results of uneluted Pb (II)-IIP@MDS; (**e**) contact angle test results of Pb (II)-IIP@MDS.

Contact angle analysis was conducted to evaluate the surface wettability and modification effects of materials at different stages. DS (Figure 1a) has a high-density Si-OH surface, interacting with water via H-bonds to yield a 28.3° contact angle. This result is consistent with the abundance of reactive Si-OH groups on the DS surface [44], providing active sites for subsequent KH550 grafting. After KH550 grafting (Equations (2) and (3)), MDS (Figure 1b) replaces Si-OH with Si-O-Si, introducing -NH_2_; silane-filled nano-pores reduce roughness, boosting hydrophobicity to 72.9°. This wetting transition directly confirms the successful grafting of KH550 onto the sand particle surface. The reduction in hydrophilic silanol groups and the introduction of hydrophobic silane chains are the direct outcomes of effective modification [45]. Post-polymerization of NHMA/HEMA (Figure 1c), a -OH/-CONH- hydrophilic layer, covers the surface. These polar groups offset KH550 hydrophobicity via H-bonding; the polymer network raises surface energy, lowering the angle to 45.3°. Pb (II) coordinates with -OH/-CONH- (Figure 1d), forming a rigid “polymer–template” structure [46]. Coordination shields hydrophilic groups, reducing H-bond sites; Pb (II)’s positive charge orders the hydration layer, hindering spreading and decreasing the angle. After template elution (Figure 1e), Pb (II)-matching cavities form. Their capillary effect accelerates water penetration; increased polar group area enhances H-bonding, minimizing the contact angle.

(2) $$H_{2}N{\left(CH_{2}\right)}_{3}Si(OC_{2}{H_{5})}_{3}+3H_{2}O\to H_{2}N(CH_{2}{)}_{3}Si(OH{)}_{3}+3C_{2}H_{5}OH$$

(3) $$H_{2}N(CH_{2}{)}_{3}Si(OH{)}_{3}+3(DS)Si-OH\to H_{2}N(CH_{2}{)}_{3}Si-(O-Si{)}_{3}+3H_{2}O$$

#### 3.1.4. FTIR Spectral Analysis

FTIR resolves material chemical structure evolution via functional group vibration detection [47]. Comparing FTIR spectra of Pb (II)-NIP@MDS, Pb (II)-IIP@MDS, and uneluted Pb (II)-IIP@MDS reveals structural changes and imprinting effects through group vibrations.

Figure S3 shows that Pb (II)-NIP@MDS has 3557.49 cm^−1^ silica stretching under low-intensity vibration reflecting reduced -OH by KH550 [48]. Pb (II)-IIP@MDS exhibits 3550.05 cm^−1^ red shift and 2977.07 cm^−1^ C-H stretch, confirming organic grafting and -OH organic interaction. Uneluted Pb (II)-IIP@MDS shows enhanced 3474.52 cm^−1^ intensity and broadening due to Pb (II)-imprint site coordination. The Pb (II)-IIP@MDS and uneluted Pb (II) from IIP@MDS which presented 2561.84cm^−1^ to 2558.88 cm^−1^ stretching is attributed to weak N-H/O-H stretching vibrations, while the C=O stretching vibration at 1722.92 cm^−1^ confirms the polymerization reaction [48]. The increased intensity at 2558.88 cm^−1^ correlates with interactions between Pb (II) and polar functional groups [49]. In 1600–550 cm^−1^ Pb (II)-NIP@MDS, Si-O vibration is masked. Pb (II)-IIP@MDS organic grafting modifies Si-O environments in uneluted sample peak splitting stems from Pb (II)-restricted organic chain vibration. Enlarged 3120–3100 cm^−1^ and 1440–1500 cm^−1^ ranges in weak Pb (II)-NIP@MDS peaks match alkyl vibrations. Pb (II)-IIP@MDS peak shifts relate to imprinted layer construction.

#### 3.1.5. SEM-EDS Complementary Characterizations

**Figure 2:**
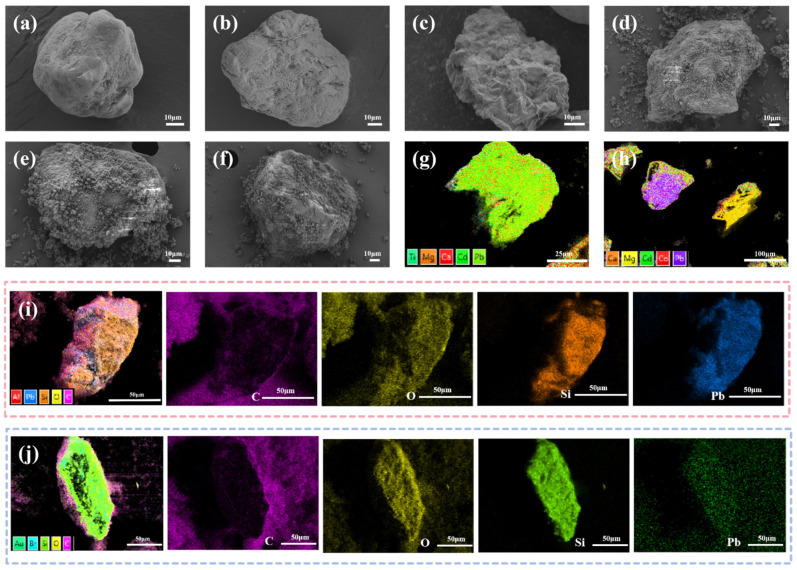
SEM and EDS characterizations of ion–imprinted materials and carriers. (**a**) Uneluted Pb (II)-IIP@MDS; (**b**) Pb (II)-IIP@MDS; (**c**) Pb (II)-IIP@MDS after multiple desorption cycles; (**d**) DS; (**e**) DS (HCl-treated); (**f**) MDS; (**g**) EDS of Pb (II)-IIP@MDS after adsorption in multi-component system; (**h**) EDS of Pb (II)-NIP@MDS after adsorption in multi-component system; (**i**) EDS element mapping of uneluted Pb (II)-IIP@MDS; (**j**) EDS element mapping of Pb (II)-IIP@MDS.

To characterize the surface morphology and elemental composition of the material, SEM-EDS analysis was performed, with the results shown in Figure 2. Figure 2a has a smooth, dense, granular surface with few active sites, unfavorable for functional monomer grafting. Figure 2b shows the surface etched by HCl consistent with XRD surface microdefects. Section 3.1.1 covers increased roughness, and acid pit etching which dissolves impurities and exposes more active sites, laying the foundation for subsequent modification. Figure 2c shows the wrinkled surface of KH550 hydrolyzed and grafted onto DS, introducing amino groups optimizing surface hydrophilicity–hydrophobicity per contact angle test. Section 3.1.3 provides chemical sites for functional monomer anchoring, making MDS a key precursor for imprinted layer construction.

Figure 2d shows a dense irregular particle layer on the surface, which is an imprinted layer formed by template ions, functional monomers, and cross-linking agents polymerization particle stacking, reflecting in situ growth characteristic of initial polymerization stage template ions that are not eluted and occupied imprinted pores [33]. Figure 2e shows a rough, porous structure with pores in the surface particle layer consistent with EDS Pb (II) signal disappearance after elution, confirming successful imprinted pores construction. Post multiple desorption cycles, Figure 2f shows that the surface retains a porous structure but local abrasion occurs.

Figure 2g shows that the element distribution is primarily Pb (II), while Figure 2h presents dispersed irregular element signals, confirming that the specific recognition of the imprinted channels significantly enhances adsorption selectivity. The strong element signals in Figure 2i correspond to the uniform distribution of functional monomers, cross-linkers, template ions, and the carrier substrate in the imprinted layer, indicating that the template ions and functional monomers were uniformly dispersed during polymerization and successfully loaded onto the silica-based MDS. The Pb (II) signal in the eluted sample (Figure 2j) shows a sharp decay, yet the distribution retains the chemical characteristics of the imprinted layer, verifying the successful elution of the template ions and the preservation of the material’s chemical composition post-elution.

#### 3.1.6. BET Test Results

To explore the micropore structural characteristics of Pb (II)-IIP@MDS and Pb (II)-NIP@MDS and clarify how pore structure affects adsorption performance, both were characterized via BET specific surface area measurement [50]. From N_2_ adsorption–desorption isotherms, key parameters were quantified, with pore size distribution patterns analyzed using the BJH model.

**Figure 3:**
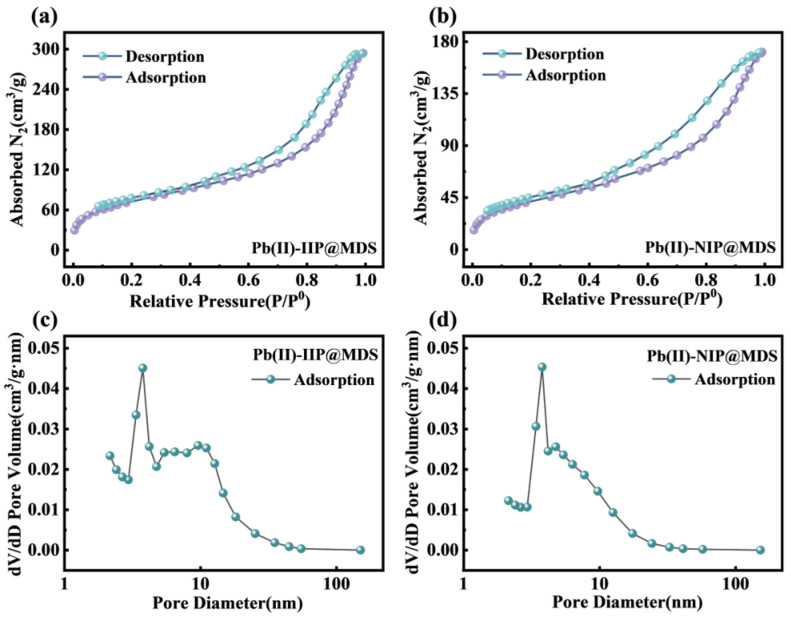
Pore structure characterization of ion-imprinted materials. (**a**) N_2_ adsorption–desorption isotherms of Pb (II)-IIP@MDS; (**b**) N_2_ adsorption–desorption isotherms of Pb (II)-NIP@MDS; (**c**) pore size distribution curves of Pb (II)-IIP@MDS; (**d**) pore size distribution curves of Pb (II)-NIP@MDS.

Figure 3a,b shows that N_2_ adsorption–desorption isotherms of Pb (II)-IIP@MDS and Pb (II)-NIP@MDS both exhibit stepped capacity increase with rising relative pressure, corresponding to a Type IV isotherm, confirming dominant mesoporous structures. At low P/P_0_ below 0.4, gentle rises correspond to N_2_ monolayer–multilayer adsorption; at high P/P_0_, sharp increases stem from mesopore capillary condensation. Pb (II)-IIP@MDS has higher overall adsorption capacity, indicating larger specific surface area and more favorable pore volume for adsorption. Figure 3c,d reveals that BJH-calculated pore size distribution curves both show unimodal distribution, with peaks at 2–5 nm further verifying mesopore dominance [51]. Pb (II)-IIP@MDS has a higher peak, suggesting a larger mesopore volume and more abundant effective adsorption sites derived from mesoporous structures. As shown in Table S1, the average pore size of Pb (II)-IIP@MDS is 7.01 nm, while the effective diameter of hydrated Pb (II) ions is approximately 0.4–0.6 nm. The significantly larger pore size of the adsorbent provides ample space for hydrated Pb (II), thereby ensuring the feasibility of mass transfer and adsorption combination.

#### 3.1.7. TGA and DTG Test Results

**Figure 4:**
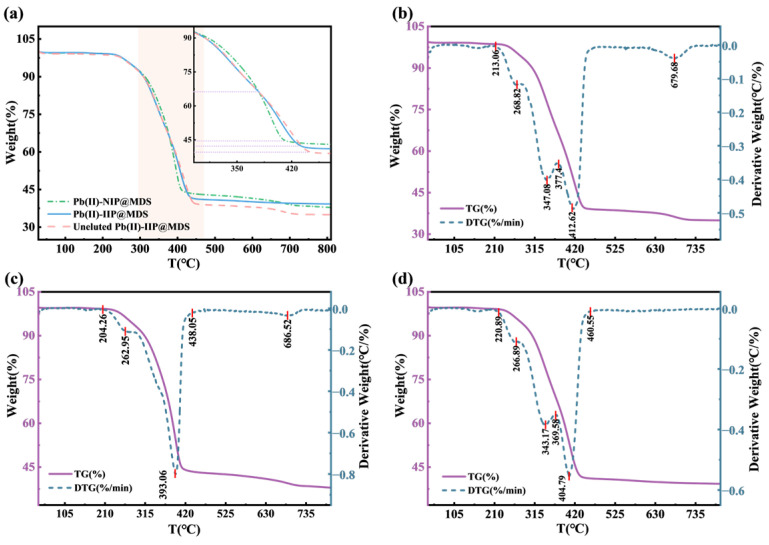
TGA characterizations of ion-imprinted materials. (**a**) TG comparison of materials in different states; (**b**) TG and DTG curves of Pb (II)-NIP@MDS; (**c**) TG and DTG curves of Pb (II)-IIP@MDS; (**d**) TG and DTG curves of uneluted Pb (II)-IIP@MDS.

TGA/DTG clarifies the thermal stability and structural evolution of ion-imprinted composites. Figure 4a shows silica as a carrier of Pb (II) that modulates thermal weight loss via organic–inorganic interface regulation. Above 600 °C, Pb (II)-NIP@MDS mass retention aligns with silica (stable Si-O bonds). Pb (II)-IIP@MDS shows 300–500 °C impacting weight loss (organic decomposition) without affecting silica stability. Template ions (in uneluted Pb (II)-IIP@MDS) act as bridges: they advance low-temperature weight loss and intensify medium-temperature loss. DTG curves in Figure 4b–d show that Pb (II)-NIP@MDS decomposition depends on whether silica dominates stability. Pb (II)-IIP@MDS has multiple peaks at 210–280 °C from monomer grafting and strong peaks at 315–420 °C from cross-linker decomposition; this reflects multicomponent organics. Uneluted Pb (II)-IIP@MDS has an intensified 200–300 °C broad peak and a shifted 300–420 °C peak; template ions enhance organic decomposition (no silica stability loss). In short, template ions reshape organic–inorganic interface interaction: thermal behavior shifts from silica-dominated stability to synergistic multi-stage response.

### 3.2. Analysis of Adsorption Experimental Results

#### 3.2.1. Influence of Multiple Factors on Adsorption Process

##### Concentration Effect

As shown in Figure S4a, with increasing initial Pb (II) concentration, Pb (II)-IIP@MDS adsorption capacity rises continuously to stabilization, while Pb (II)-NIP@MDS shows a slight increase and low saturation. Due to ion imprinting, Pb (II)-IIP@MDS has matching surface sites that fully bind Pb (II) with concentration rise, achieving concentration-driven saturation. Pb (II)-NIP@MDS, lacking specific sites, has weak binding capacity and reaches adsorption limit at low concentrations.

##### Time Effect

As shown in Figure S4b, Pb (II)-IIP@MDS adsorption capacity rises rapidly with time, reaching equilibrium after 120 min. Initial abundant surface vacancies enable fast Pb (II) diffusion/binding; later, fewer vacancies and higher mass transfer resistance slow the rate to equilibrium [52]. For practical use, 120 min adsorption time balances efficiency, reduces unnecessary energy consumption, and boosts process economy.

##### pH Effect

As shown in Figure S4c, Pb (II)-IIP@MDS adsorption capacity rises, then falls, with pH, peaking at pH 6–7. At low pH, high H^+^ concentration competes with Pb (II) for adsorption sites, inhibiting adsorption. With increasing pH, reduced H^+^ enhances site affinity for Pb (II), raising adsorption capacity. However, excessive pH (>7) causes Pb (II) hydrolysis into Pb (OH)_n_ precipitates, lowering free Pb (II) concentration and reducing adsorption [53]. Practical operation needs precise pH control at 6–7 to balance efficiency and hydrolysis risk, ensuring process stability.

##### Temperature Effect

Within the range of Figure S4d, increasing temperature raises Pb (II)-IIP@MDS adsorption capacity and shortens equilibrium time. This is because a higher temperature accelerates Pb (II) diffusion and favors endothermic adsorption (Table S4, ∆H^0^ > 0) by further promoting a reaction, thus increasing capacity.

#### 3.2.2. Adsorption Isotherm Models

The Langmuir model assumes monolayer adsorption with uniform, non-interacting sites. The Freundlich model is based on multilayer adsorption with heterogeneous sites. The equations are given in Equations (4) and (5). Comparing fitting parameters (the relevant parameters presented in Table S2) identifies adsorption mechanism and potential for Pb (II).

(4) $$Q_{e}=\frac{Q_{m}C_{e}K_{L}}{1+K_{L}C_{e}}$$

(5) $$Q_{e}=K_{F}C_{e}^{\frac{1}{n}}$$

Among them, $K_{L}$ and $K_{F}$ are the adsorption equilibrium constants for the Langmuir and Freundlich models, respectively; $Q_{m}$ is the theoretical maximum adsorption capacity constant; and $\frac{1}{n}$ is the adsorption intensity index. All these parameters are used to quantitatively analyze adsorption isotherm behavior.

**Figure 5:**
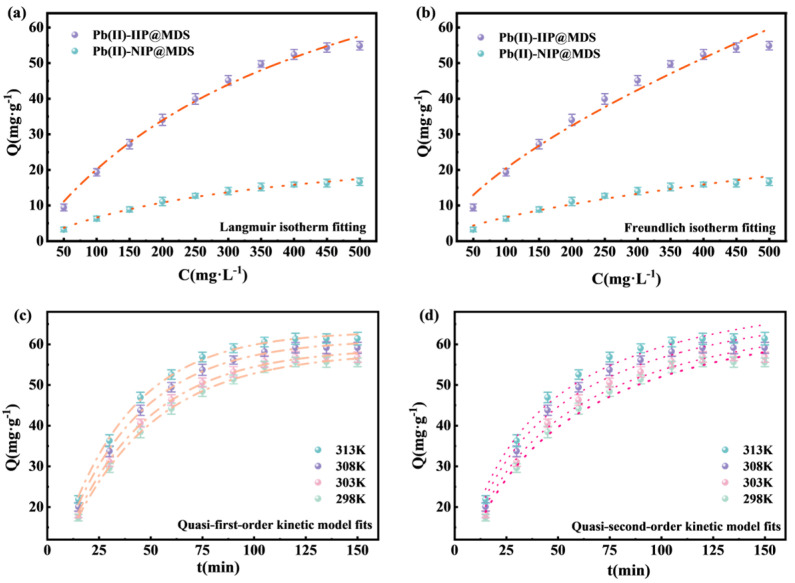
(**a**) Langmuir isotherm model fitting curves for Pb (II) adsorption by Pb (II)-IIP@MDS and Pb (II)-NIP@MDS; (**b**) Freundlich isotherm model fitting curves for Pb (II) adsorption by Pb (II)-IIP@MDS and Pb (II)-NIP@MDS; (**c**) quasi-first-order kinetic model fitting curves for Pb (II) adsorption by Pb (II)-IIP@MDS; (**d**) quasi-second-order kinetic model fitting curves for Pb (II) adsorption by Pb (II)-IIP@MDS.

Figure 5a shows that the adsorption capacity of Pb (II)-IIP@MDS rapidly increases to saturation with increasing concentration, while that of Pb (II)-NIP@MDS exhibits a lower adsorption capacity. Ion imprinting endows Pb (II)-IIP@MDS with single-layer sites matching Pb (II), exhibiting saturation concentration consistent with the Langmuir model (R^2^ = 0.992). In contrast, Pb (II)-NIP@MDS lacks specific sites, relying on non-specific weak interactions with low saturation. Figure 5b shows R^2^ values of 0.972 and 0.96 for Pb (II)-IIP@MDS and Pb (II)-NIP@MDS, respectively, both below the Langmuir value. The Freundlich model, applicable to heterogeneous site adsorption, indicates trace multilayer nonspecific interactions. However, Pb (II)-IIP@MDS is primarily dominated by imprinted sites, resulting in a weaker Freundlich fit that further confirms its monolayer-specific adsorption characteristics. In summary, Pb (II)-IIP@MDS adsorption is dominated by Langmuir monolayer chemisorption, with a secondary contribution from multilayer adsorption as indicated by the Freundlich model.

#### 3.2.3. Adsorption Kinetic Models

The quasi-first-order model assumes adsorption rate controlled by adsorbate diffusion between solution and adsorbent surface, applying to physical adsorption-dominated processes. The quasi-second-order model is based on chemisorption dominance with rate controlled by chemical interactions between surface active sites and adsorbate, better fitting adsorption involving chemical bonding. Comparing fitting parameters in Table S3 identifies the rate-limiting step and adsorption nature; the equations are given in Equations (6) and (7).

(6) $$\ln\left(Q_{e}-Q_{t}\right)=lnQ_{e}-k_{1}t$$

(7) $$\frac{t}{Q_{t}}=\frac{k_{2}}{Q_{e}^{2}}+\frac{t}{Q_{e}}$$

Among them, $Q_{e}$ and $Q_{t}$ represent the calculated adsorption capacities at equilibrium and time t (mg·g^−1^), respectively; $k_{1}$ and $k_{2}$ are the rate constants.

Figure 5c shows that adsorption capacity rises rapidly then stabilizes with time across temperatures, with high linearity of fitting curves. A higher temperature accelerates equilibrium and increases capacity: initial abundant surface vacancies enable fast adsorbate diffusion and site occupation, while fewer vacancies and increased diffusion resistance later slow the rate to equilibrium. A higher temperature intensifies molecular thermal motion, accelerating adsorbate diffusion and site binding to promote faster equilibrium and higher capacity.

Yet the quasi-first-order model alone cannot confirm chemical interactions, requiring further analysis with the quasi-second-order model. Figure 5d presents a similar time-dependent trend: higher temperatures accelerate equilibrium and increase capacity. Initially, physical diffusion brings Pb (II) to the adsorbent surface, and ion-imprinted sites rapidly chemically interact with Pb (II) to accelerate capacity rise. Later, as sites fill, chemical interaction driving force decreases, slowing the rate to equilibrium.

In this study, although the quasi-second-order model has a slightly lower but still high R^2^, its fitting logic (chemically controlled) aligns better with the adsorption nature, considering the “ion imprinting” characteristic. This indicates that the adsorption process is not dominated by a single mechanism but a synergy of “diffusion” and “chemical interaction” [42].

#### 3.2.4. Adsorption Thermodynamic Models

Adsorption spontaneity and energy-driven mechanisms are revealed via thermodynamic functions. The Van’t Hoff equation and Gibbs free energy change formula explain adsorption thermodynamic behavior, determining thermal effects, entropy changes, and spontaneous directions with parameters in Table S4; the equations are given in Equations (8) and (9).

(8) $$lnK=-\frac{∆H^{0}}{RT}+\frac{∆S^{0}}{R}$$

(9) $$\Delta G^{0}=-RTlnK$$

Among them, *∆G*^0^ is the standard molar Gibbs free energy change (kJ·mol^−1^); *∆S*^0^ is the standard molar entropy change (J·mol^−1^·K^−1^); *∆H*^0^ is the adsorption enthalpy change (kJ·mol^−1^); and *R* is the gas constant.

Figure S5 and Table S4 show Van’t Hoff fitting curves for Pb (II) adsorption by Pb (II)-IIP@MDS with R^2^ = 0.96 indicating high linear correlation between ln(K) and 1/T. The curve has a negative slope combined with ∆H^0^ > 0, increasing temperature, decreasing 1/T, and raising ln(K), namely higher adsorption equilibrium constant K. This means that higher temperature favors adsorption, consistent with ∆G^0^ becoming more negative, with temperature confirming the thermodynamic characteristic of adsorption promoted by elevated temperature. ∆S^0^ > 0 arises from Pb (II) transferring from liquid phase to Pb (II)-IIP@MDS surface binding specifically to imprinted sites and releasing solvent molecules, which increases free molecules in solution and raises disorder.

With ∆H^0^ > 0 and ∆S^0^ > 0, ∆G^0^ becomes more negative as temperature rises—indicating that Pb (II) adsorption by Pb (II)-IIP@MDS is an endothermic, entropy-increasing, and spontaneous process [54]. The activation energy for Pb (II) adsorption on Pb (II)-IIP@MDS was calculated to be 13.72 kJ·mol^−1^ (via Arrhenius equation), indicating a physical–chemical mixed adsorption process with moderate binding strength between the adsorbent and Pb (II) ions.

### 3.3. Selective Adsorption

**Figure 6:**
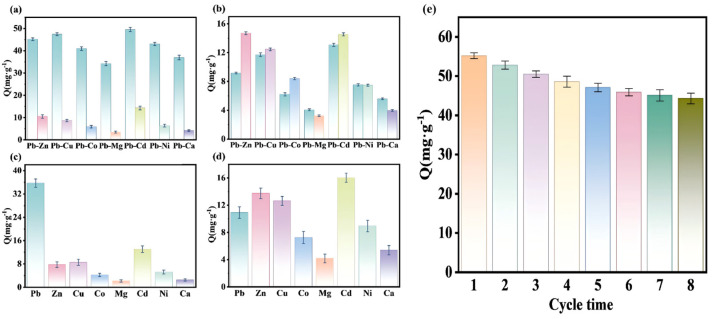
(**a**,**b**) Selectivity of Pb (II)-IIP@MDS and Pb (II)-NIP@MDS for Pb (II) in binary system; (**c**,**d**) selectivity of Pb (II)-IIP@MDS and Pb (II)-NIP@MDS for Pb (II) in multi-component system; (**d**) Pb (II)-IIP@MDS characterizations of repeated adsorption cycle performance; (**e**) repeated adsorption–desorption cycle performance of Pb (II)-IIP@MDS.

Figure 6a,c demonstrates that in both binary and multicomponent systems, the adsorption capacity of Pb (II)-IIP@MDS for Pb (II) is significantly higher than that for other heavy metals, reflecting the ion-imprinting effect. The error bars in the Figures represent the standard deviation of three parallel replicates to reflect data reliability. To quantify the selectivity, three key evaluation parameters were used (consistent with Table S5). Selectivity tests were conducted in triplicate at 25 ± 1 °C and pH 7.0 (adjusted with 0.1 M HCl/NaOH), with equilibrium confirmed through kinetic studies. K_sp_ calculations confirmed no metal hydroxide precipitation. As shown in Table S5, the partition coefficient (K_d_) of Pb (II)-IIP@MDS for Pb (II) is significantly higher than that for competing ions, and the selectivity coefficient (a) of Pb (II) relative to Mg^2+^ reaches 14.53, markedly higher than that of the Pb (II)-NIP@MDS system. This selectivity coefficient is comparable to or higher than those previously reported for Pb (II)-IIPs, confirming the competitive selectivity of this material [55,56]. The pre-assembled imprinted cavity for template Pb (II) enables specific matching recognition of Pb (II) [57]; other heavy metals cannot fit this cavity structure, severely limiting their adsorption capacity. Figure 6b,d indicate that Pb (II)-NIP@MDS without specific imprinted cavities primarily relies on non-specific surface interactions. The distribution coefficient (K_d_) of Pb (II) falls within the range of competing ions (see Table S5), with adsorption capacity fluctuating according to the charge and ionic radius of heavy metal ions. Consequently, Cd^2+^ and Cu^2+^ with higher charge densities exhibit stronger adsorption than Ca^2+^ and Mg^2+^, and even their coexisting ion adsorption capacities exceed that of Pb (II).

### 3.4. Reusability

Figure 6e illustrates the adsorption capacity variation in Pb (II)-IIP@MDS over eight adsorption cycles, reflecting its reuse performance trend. The adsorption capacity generally decreased with increasing cycle number. This decay resulted from partial irreversible occupation of imprinted sites and slight micropore shrinkage (verified by SEM image Figure 2f after cycling), leading to a reduction in accessible active sites. This represents a typical decay trend for imprinted polymers during repeated elution–adsorption cycles. Nevertheless, the adsorbent retained 80.3% of its initial efficiency after eight cycles, a value comparable to or higher than most reported Pb (II)-imprinted polymers under similar conditions.

### 3.5. Imprinting and Adsorption Mechanisms

#### 3.5.1. Imprinting and Adsorption Mechanism

**Figure 7:**
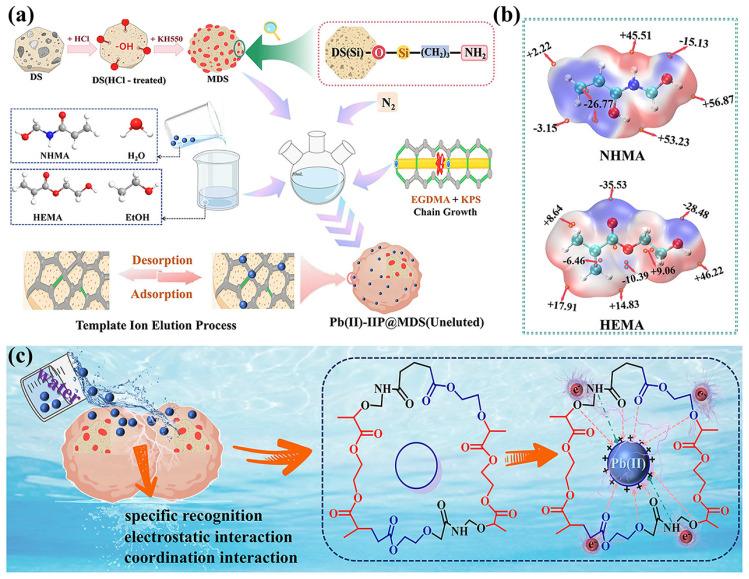
(**a**) Preparation process of Pb (II)-IIP@MDS; (**b**) ESP (electrostatic potential) of NHMA and HEMA; (**c**) adsorption process mechanism of Pb (II)-IIP@MDS.

Pb (II)-IIP@MDS preparation starts with functional monomers (NHMA/HEMA) pre-assembled with template Pb (II): ESP calculations are performed using the Amsterdam Modeling Suite (AMS) (SCM, The Netherlands). ESP analysis (Figure 7b) shows that NHMA’s -CONH-/-CH_2_OH and HEMA’s C=O/-OH (negatively charged regions) coordinate/electrostatically interact with positive Pb (II), forming an ordered pre-assembled complex [58]. This provides the chemical basis for imprinted cavity compatibility.

EGDMA (cross-linker) and KPS (initiator) trigger free radical polymerization on MDS (Figure 7a), cross-linking monomers into a 3D network that solidifies the pre-assembled structure. Template elution forms cavities matching Pb (II) in size and retains -CONH-/-OH groups.

The adsorption process relies on cavity-specific recognition + electrostatic/coordination interactions + hydrogen bonding: weakly negative -OH/-CH_2_OH groups form hydrogen bonds with water, regulating Pb (II) hydration to promote migration to cavities (Figure 7c), while cavity matching enables stable binding.

#### 3.5.2. XPS Verification

**Figure 8:**
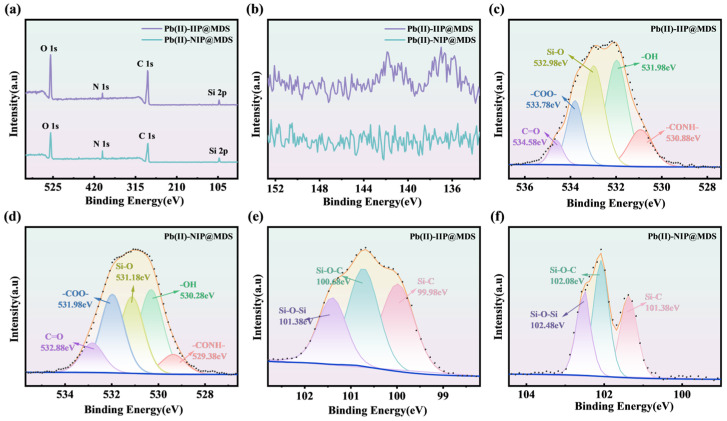
(**a**) Full-spectrum XPS of Pb (II)-IIP@MDS and Pb (II)-NIP@MDS; (**b**) Pb 4f XPS spectra of Pb (II)-IIP@MDS and Pb (II)-NIP@MDS; (**c**) O 1s XPS spectrum of Pb (II)-IIP@MDS; (**d**) O 1s XPS spectrum of Pb (II)-NIP@MDS; (**e**) Si 2p XPS spectrum of Pb (II)-IIP@MDS; (**f**) Si 2p XPS spectrum of Pb (II)-NIP@MDS.

XPS characterization (Figure 8) confirms the proposed imprinting and adsorption mechanism. Gou et al. [59] demonstrated that Pb (II) can form stable coordination bonds with -OH, -COOH, and -CONH- groups, as evidenced by shifts in the O 1s peak and characteristic Pb 4f peak positions. The O 1s spectra (Figure 8c,d) reveal ~0.5 eV shifts in -CONH-/-OH peaks and intensity changes in Pb (II)-IIP@MDS compared to Pb (II)-NIP@MDS, indicating coordination interactions between NHMA/HEMA functional groups and Pb (II) during polymerization; this alters the electron density of oxygen atoms, whereas the O 1s peak of Pb (II)-NIP@MDS aligns with the intrinsic state of the MDS support, reflecting template-free random monomer interactions. The Si 2p spectra of Pb (II)-IIP@MDS (Figure 8e,f) reveal enhanced Si-O-C and Si-C peaks, confirming successful grafting of monomers onto the MDS surface via Si-O bonds and their directed pre-assembly guided by the Pb (II) template. The Pb 4f spectrum (Figure 8b) reveals characteristic Pb (II) peaks for Pb (II)-IIP@MDS, indicating high-affinity coordination sites retained on the cavity walls after elution. In contrast, Pb (II)-NIP@MDS exhibits only baseline noise in this region, verifying the specific affinity of the imprinted cavity for Pb (II).

In summary, template-directed Pb (II) guided the oriented pre-assembly of NHMA and HEMA monomers on a sandy carrier. Following polymerization and curing, an imprinted cavity with specific coordinating residues formed, enabling selective recognition and binding of Pb (II) through matching its electronic structure and spatial configuration.

## 4. Conclusions

This study proposes a novel design strategy of innovative carrier application synergistic coordination of functional monomers’ precise construction of ion imprinting, successfully fabricating the Pb (II)-imprinted composite Pb (II)-IIP@MDS by combining common desert sand as carrier NHMA and HEMA functional monomers and Pb (II) as a template ion via ion-imprinting polymerization. BET, FTIR, XPS, and SEM-EDS systematically validated the imprinting effect and provided experimental support for the proposed adsorption mechanism. Kinetic and thermodynamic results showed that the adsorption follows the Langmuir isotherm and quasi-first- and second-order models with synergistic control of physical diffusion and chemical interaction, and equilibrium achieved at 120 min under thermodynamic parameters ΔH^0^ > 0 ΔS^0^ > 0 ΔG^0^ < 0 confirmed that the process is endothermic, spontaneous, and entropy-increasing. In competitive systems, Pb (II)-IIP@MDS exhibited superior Pb (II) selectivity with maximum selectivity coefficient 14.53, compared to non-imprinted Pb (II)-NIP@MDS, and maintained 80.3% adsorption efficiency after eight adsorption–desorption cycles demonstrating good structural stability and regenerability. This work offers practical low-cost materials and a theoretical basis for deep purification of Pb (II)-containing heavy metal wastewater.

## Data Availability

The original contributions presented in this study are included in the article/Supplementary Material. Further inquiries can be directed to the corresponding author.

## Supplementary Materials

The following supporting information can be downloaded at: https://www.mdpi.com/article/10.3390/polym18010042/s1.
