# Supplementary material for: Preparation, Adsorption Performance and Mechanism of Low-Cost Desert Sand-Based Pb (II) Ion-Imprinted Composites
Source: Polymers (Basel). 2025 Dec 23;18(1):42. doi: 10.3390/polym18010042 (PMC12787551; doi:10.3390/polym18010042)
Supplement: Supplementary file 1 [file polymers-18-00042-s001.zip › polymers-4038424-supplementary.pdf]

Supporting Information

Preparation, Adsorption Performance and Mechanism of Low-Cost Desert Sand-Based Pb(II) Ion-Imprinted Composites

Yixin Sui, Jiaxiang Qi, Shuaibing Gao, Linlin Chai, Yahong Xie, Changyan Guo, Shawket Abliz *

Key Laboratory of Oil and Gas Fine Chemicals of Ministry of Education, School of Chemical Engineering, Xinjiang University, Urumqi 830017, China

* Corresponding author address: School of Chemical Engineering, Xinjiang University, Urumqi 830017, China

Email: shawket_abliz@sina.com

Figure captions:

Fig. S1. (a) XRD patterns of XDS and DS (HCl - treated); (b) Enlarged views of characteristic diffraction peaks.

Fig. S2. (a) UV spectra of functional monomer proportion; (b) magnified view of monomer proportion; (c) UV spectra of dual-functional monomer blending ratio; (d) UV spectra of monomer-template complex in the pre-fabrication stage of ion-imprinted composites.

Fig. S3. (a) Full - range FTIR spectra comparison of Pb(II)-NIP@MDS, Pb(II)-IIP@MDS, and Uneluted Pb(II)-IIP@MDS; (b) Enlarged spectra of characteristic peaks. All spectra were baseline-corrected and vertically offset for clarity.

Fig. S4. (a) Effects of initial concentration (C, mg · L^{-1}) on Pb(II)-IIP@MDS and Pb(II)-NIP@MDS; (b) Effects of adsorption time (t, min) on adsorption capacity of Pb(II)-IIP@MDS; (c) Effects of pH on adsorption capacity of Pb(II)-IIP@MDS; (d) Effects of temperature (298K, 303K, 308K, 313K) on adsorption kinetics of Pb(II)-IIP@MDS

Fig. S5. (a) Van't Hoff fitting curves for the thermodynamics of Pb(II) adsorption by Pb(II)-IIP@MDS; (b)Arrhenius fitting curve for the adsorption thermodynamics of Pb(II) on Pb(II)-IIP@MDS

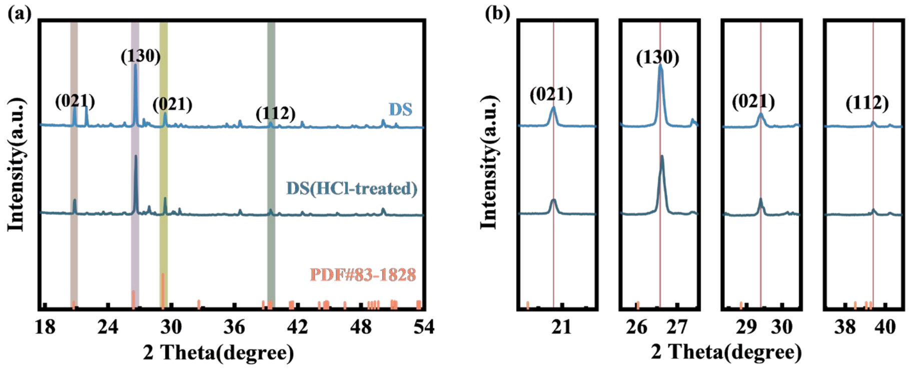
(a)
(b)
(021)
(130)
(021)
(112)
DS
DS(HCl-treated)
PDF#83-1828
Intensity(a.u.)
2 Theta(degree)
18 24 30 36 42 48 54
21 26 27 29 30 38 40

Fig. S1.

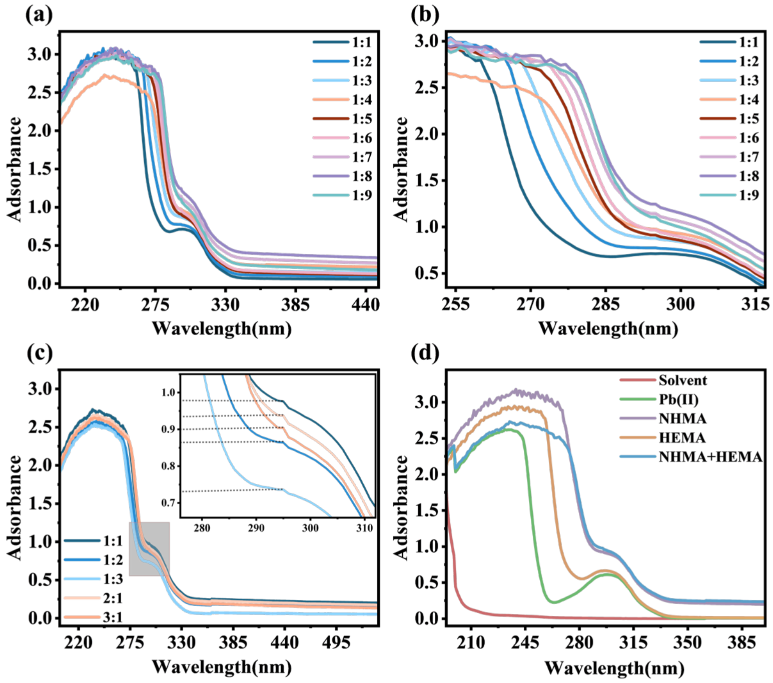
(a)
(b)
(c)
(d)
Adsorbance
Wavelength(nm)
1:1
1:2
1:3
1:4
1:5
1:6
1:7
1:8
1:9
2:1
3:1
Solvent
Pb(II)
NHMA
HEMA
NHMA+HEMA

Fig. S2.

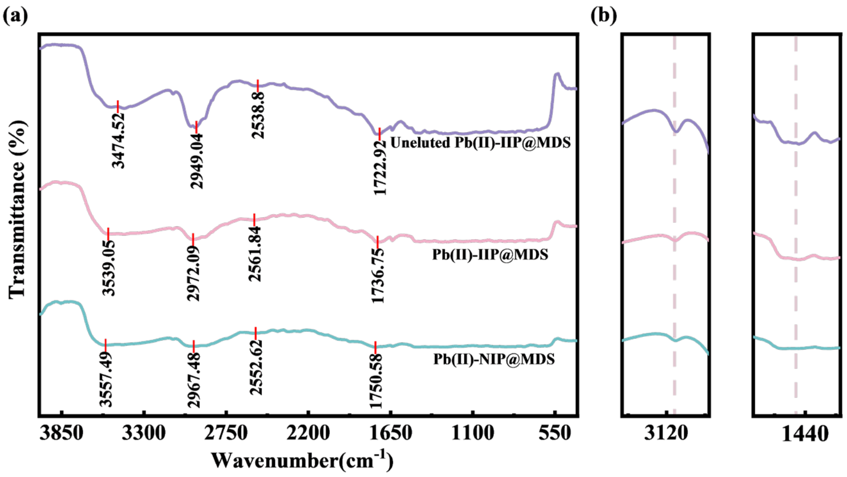
(a)
(b)
Transmittance (%)
3474.52
2949.04
2538.8
1722.92
Uneluted Pb(II)-IIP@MDS
3539.05
2972.09
2561.84
1736.75
Pb(II)-IIP@MDS
3557.49
2967.48
2552.62
1750.58
Pb(II)-NIP@MDS
3850
3300
2750
2200
1650
1100
550
Wavenumber(cm-1)
3120
1440

Fig. S3.

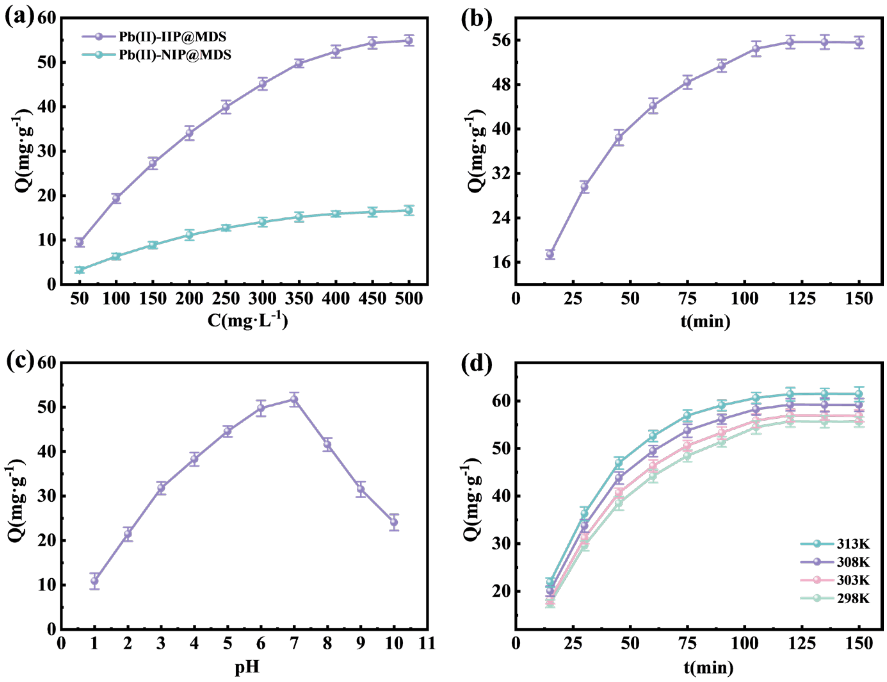
(a)
Pb(II)-IIP@MDS
Pb(II)-NIP@MDS
Q(mg·g-1)
C(mg·L-1)
(b)
t(min)
(c)
pH
(d)
313K
308K
303K
298K

Fig. S4.

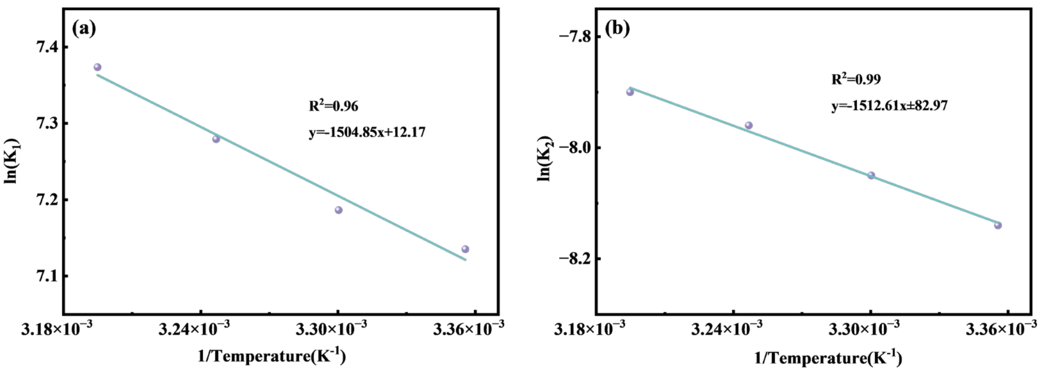

(a)
7.4
7.3
7.2
7.1
ln(K1)
R2=0.96
y=-1504.85x+12.17
3.18×10−3
3.24×10−3
3.30×10−3
3.36×10−3
1/Temperature(K-1)
(b)
−7.8
−8.0
−8.2
ln(K2)
R2=0.99
y=-1512.61x±82.97
3.18×10−3
3.24×10−3
3.30×10−3
3.36×10−3
1/Temperature(K-1)

Fig. S5.

Table S1. Pore structure parameters of Pb(II)-IIP@MDS and Pb(II)-NIP@MDS

Sample	Specific Surface Area ($m^2 \cdot g^{-1}$)	Pore Volume ($cm^3 \cdot g^{-1}$)	Average Pore Diameter(nm)
Pb(II)-IIP@MDS	252.18	0.26	7.01
Pb(II)-NIP@MDS	157.78	0.46	6.81

Table S2. Isotherm model fitting parameters for Pb(II) adsorption by Pb(II)-IIP@MDS and Pb(II)-NIP@MDS

**Adsorbent**	**Langmuir Isotherm** [a]			**Freundlich Isotherm** [b]		
	**K_L**	**Q_m($mg \cdot g^{-1}$)**	**R^2**	**K_F**	**1/n**	**R^2**
Pb(II)-IIP@MDS	$2.31*10^{-3}$	107.44	0.992	0.955	0.665	0.972
Pb(II)-NIP@MDS	$2.87*10^{-3}$	29.59	0.994	0.380	0.623	0.969

[a] *Langmuir Isotherm:*

$$Q_e = \frac{Q_m C_e K_L}{1+K_L C_e}$$

[b] *Freundlich Isotherm:*

$$Q_e = K_F C_e^{\frac{1}{n}}$$

Among them, K_L and K_F are the adsorption equilibrium constants for the Langmuir and Freundlich models, respectively; Q_m is the theoretical maximum adsorption capacity constant; and $\frac{1}{n}$ is the adsorption intensity index. All these parameters are used to quantitatively analyze the adsorption isotherm behavior.

Table S3. Kinetic model fitting parameters for Pb(II) adsorption by Pb(II)-IIP@MDS

T (K)	Quasi-First-Order [c]			Quasi-Second-Order [d]		
	$Q_{e,cal}$ mg·g^{-1}	k_1 min^{-1}	R^2	$Q_{e,cal}$ mg·g^{-1}	K_2 min^{-1}	R^2
298k	58.10	$2.40*10^{-2}$	0.998	75.65	$2.91*10^{-4}$	0.991
303k	59.11	$2.54*10^{-2}$	0.998	75.87	$3.18*10^{-4}$	0.990
308k	61.18	$2.74*10^{-2}$	0.999	77.67	$3.47*10^{-4}$	0.985
313k	63.22	$2.93*10^{-2}$	0.999	79.52	$3.69*10^{-4}$	0.982

[c] *Quasi-First-Order:*

$$\ln(Q_e - Q_t) = lnQ_e - k_1 t$$

[d] *Quasi-Second-Order:*

$$\frac{t}{Q_t} = \frac{k_2}{Q_e^2} + \frac{t}{Q_e}$$

Among them, Q_e and Q_t represent the calculated adsorption capacities at equilibrium and time t (mg·g^{-1}), respectively; k_1 and k_2 are the rate constants.

Table S4. Thermodynamic parameters for the adsorption of Pb(II) by Pb(II)-IIP@MDS

ΔH^0(kJ·mol^{-1})	ΔS^0(J·mol^{-1}·K^{-1})	ΔG^0 (kJ·mol−1)			
		298K	303K	308K	313K
12.51	101.19	-17.68	-18.10	-18.64	-19.19

[e] *Van't Hoff equation:*

$$lnK = -\frac{\Delta H^0}{RT} + \frac{\Delta S^0}{R}$$

[f] *Gibbs free energy change formula:*

$$\Delta G^0 = -RTlnK$$

Among them, ΔG^0 is the standard molar Gibbs free energy change (kJ·mol^{-1}); ΔS^0 is the standard molar entropy change (J·mol^{-1}·K^{-1}); ΔH^0 is the adsorption enthalpy change (kJ·mol^{-1}); and R is the gas constant.

Table S5. Distribution coefficients (kd), selectivity coefficients (α), and relative selectivity coefficients (K) of Pb(II)-IIP@MDS and Pb(II)-NIP@MDS for different heavy metal ions

M(II)	Pb(II)-IIP@MDS			Pb(II)-NIP@MDS			K
	$k_{d,\ Pb(II)}$	$k_{d,\ M(II)}$	a	$k_{d,\ Ni(II)}$	$k_{d,\ M(II)}$	a	
Zn(II)	0.825	0.118	6.99	0.101	0.172	0.59	11.95
Cu(II)	0.903	0.095	9.49	0.133	0.142	0.93	16.21
Co(II)	0.694	0.063	10.99	0.066	0.092	0.72	15.20
Mg(II)	0.521	0.036	14.53	0.042	0.033	1.27	11.46
Cd(II)	0.982	0.167	5.87	0.151	0.170	0.89	6.62
Ni(II)	0.757	0.068	11.10	0.082	0.081	1.01	11.00
Ca(II)	0.587	0.043	13.64	0.059	0.041	1.43	9.57

[g] *distribution ratio:*

$$K_d = Q_e/C_e$$

[h] *selectivity coefficient:*

$$a = K_{d,Pb(II)}/K_{d,M(II)}$$

[I] *relative selectivity coefficient:*

$$K = a_{Pb}^{IIP}/a_{Pb}^{NIP}$$

Herein, $M(II)$ represents the competing ion; a_{Pb}^{IIP} and a_{Pb}^{NIP} denote the selectivity coefficients of Pb(II)-IIP@MDS and Pb(II)-NIP@MDS towards Pb(II), respectively.
